# Arabidopsis KASH Proteins SINE1 and SINE2 Are Involved in Microtubule Reorganization During ABA-Induced Stomatal Closure

**DOI:** 10.3389/fpls.2020.575573

**Published:** 2020-11-20

**Authors:** Alecia Biel, Morgan Moser, Iris Meier

**Affiliations:** ^1^Department of Molecular Genetics, The Ohio State University, Columbus, OH, United States; ^2^Center for RNA Biology, The Ohio State University, Columbus, OH, United States

**Keywords:** KASH, nuclear envelope, SINE proteins, microtubules, abscisic acid

## Abstract

Abscisic acid (ABA) induces stomatal closure by utilizing complex signaling mechanisms, allowing for sessile plants to respond rapidly to ever-changing environmental conditions. ABA regulates the activity of plasma membrane ion channels and calcium-dependent protein kinases, Ca^2+^ oscillations, and reactive oxygen species (ROS) concentrations. Throughout ABA-induced stomatal closure, the cytoskeleton undergoes dramatic changes that appear important for efficient closure. However, the precise role of this cytoskeletal reorganization in stomatal closure and the nature of its regulation are unknown. We have recently shown that the plant KASH proteins SINE1 and SINE2 are connected to actin organization during ABA-induced stomatal closure but their role in microtubule (MT) organization remains to be investigated. We show here that depolymerizing MTs using oryzalin can restore ABA-induced stomatal closure deficits in *sine1-1* and *sine2-1* mutants. GFP-MAP4-visualized MT organization is compromised in *sine1-1* and *sine2-1* mutants during ABA-induced stomatal closure. Loss of SINE1 or SINE2 results in loss of radially organized MT patterning in open guard cells, aberrant MT organization during stomatal closure, and an overall decrease in the number of MT filaments or bundles. Thus, SINE1 and SINE2 are necessary for establishing MT patterning and mediating changes in MT rearrangement, which is required for ABA-induced stomatal closure.

## Introduction

Stomata open and close to regulate gas exchange between the plant and its environment. These stomatal dynamics are controlled by a variety of environmental factors, including biotic and abiotic stresses. The plant hormone abscisic acid (ABA) is involved in the response to abiotic stress, including drought, salinity, temperature, and light (Zhang et al., 2008; Xi et al., 2010; Verma et al., 2016). In guard cells, ABA initiates a signaling cascade, which leads to increased H_2_O_2_ and Ca^2+^ levels and involves the reorganization of the actin cytoskeleton (Umezawa et al., 2010; Zhao et al., 2011, 2016; Jiang et al., 2012; Li et al., 2014).

We have shown previously that in Arabidopsis SUN-interacting nuclear envelope protein 1 and 2 (SINE1 and SINE2), components of a plant Linker of Nucleoskeleton and Cytoskeleton (LINC) complex, play a role in stomatal opening and closing (Biel et al., 2020). LINC complexes are protein complexes that span both the outer nuclear membrane (ONM) and inner nuclear membrane (INM) through binding between the ONM Klarsicht/ANC-1/Syne Homology (KASH) proteins, and the INM Sad1/Unc-84 (SUN) proteins. KASH proteins, which have variable cytoplasmic domains, interact directly or indirectly with a variety of cytoskeletal elements (Starr and Fridolfsson, 2010; Bone and Starr, 2016). SINE1 and SINE2 are plant KASH proteins, which bind to Arabidopsis SUN1 and SUN2 (Zhou et al., 2014). SINE1 associates with F-actin and is—in leaves—predominantly expressed in the guard cell lineage, while SINE2 is ubiquitously expressed in leaves (Zhou et al., 2014). Loss of SINE1 or SINE2 results in ABA hyposensitivity and impaired stomatal dynamics but does not affect stomatal closure induced by the bacterial elicitor flg22. The ABA-induced stomatal closure phenotype is, in part, attributed to impairments in Ca^2+^ and F-actin regulation (Biel et al., 2020).

Here, we show that SINE1 and SINE2 are also functionally related to the microtubule (MT) cytoskeleton. Guard cells have a unique radial organization pattern of the cortical MTs (Galatis and Apostolakos, 2004). Early studies of the role of MTs during stomatal dynamics came to contradictory results (Assmann and Baskin, 1998; Fukuda et al., 1998). It has now been established that MTs are essential for guard cell function (Eisinger W. et al., 2012; Eisinger W. R. et al., 2012; Jiang et al., 2014). Utilizing the MT marker GFP-TUB6 in Arabidopsis, a strong positive correlation between the number of MT filaments and stomatal aperture has been found, with ABA-induced disruption of MTs closely associated with stomatal closure (Eisinger W. et al., 2012; Eisinger W. R. et al., 2012; Jiang et al., 2014). Upon guard cell closure, remaining MTs were fewer in number and there was an increase in cytosolic fluorescence, however, remaining MTs appeared radial and total fluorescence was significantly decreased. In an accompanying paper (Eisinger W. R. et al., 2012), the authors used RFP-tagged end binding protein 1 (EB1) to show that the number of MT growing ends remained constant throughout this rearrangement. Thus, existing MTs undergo a rearrangement that facilitates MT bundling during closure, possibly in addition to MT instability (Eisinger W. et al., 2012; Eisinger W. R. et al., 2012). The inability to degrade tubulin was linked with impaired stomatal closure while the use of a MT depolymerizing drug rescued this phenotype, indicating the importance of MT reorganization in mediating closure (Khanna et al., 2014). Lastly, the plant growth regulator 5-aminolevulinic acid (ALA) inhibits stomatal closure and impairs ABA-induced stomatal closure (An et al., 2020). Drug-induced MT depolymerization alone was insufficient in inducing stomatal closure but the combination of depolymerizing MTs in the presence of ABA was able to enhance stomatal closure, further highlighting the role of MT reorganization in ABA-induced guard cell closure (An et al., 2020).

We used similar methods as Eisinger W. et al. (2012) to investigate the role of SINE1 and SINE2 in regulating MT organization during ABA-induced stomatal closure. Importantly, we show that depolymerizing MTs with oryzalin can rescue the ABA hyposensitivity of SINE1 and SINE2 mutants during ABA-induced stomatal closure. We then present evidence that SINE1 and SINE2 are required for the reorganization of the guard cell MT cytoskeleton during stomatal dynamics and present a working model for the possible role of this reorganization. Together, our data suggest that an aberrant MT organization based on the loss of either SINE1 or SINE2 is involved in the inhibition of ABA-induced stomatal closure.

## Materials and Methods

### Plant Material

*Arabidopsis thaliana* (ecotype Col-0) was grown at 23°C in soil under 16 h light and 8 h dark conditions. For all assays, rosette leaves were collected from 3 to 4 week-old Arabidopsis plants grown under these conditions. *sine1-1* (SALK_018239C) and *sine2-1* (CS801355), were previously reported and shown to have no full-length SINE1 or SINE2 mRNA accumulates, respectively (Zhou et al., 2014). 35Spro::GFP-MAP4 in Col-0 (GFP-MAP4) was obtained from Dr. Charlie Andersen at Pennsylvania State University and has been described previously (Marc et al., 1998). GFP-MAP4 was crossed with *sine1-1* or *sine2-1* and bred until homozygous *sine1-1* and *sine2-1* mutants expressing GFP-MAP4 were obtained. Genotyping for the *sine1-1* and *sine2-1* insertion alleles was performed as described before (Zhou et al., 2014).

### Stomatal Aperture Measurements

Stomatal bioassays were performed as previously described (Biel et al., 2020). Briefly, rosette leaves of 3–4 week-old plants were placed abaxial side up in opening buffer (OB) containing 10 mM MES, 20 μM CaCl_2_, 50 mM KCl, and 1% sucrose at pH 6.15 for 2 h under constant light. Leaves remained whole until designated time points at which abaxial epidermal strips were peeled and imaged using a confocal microscope (Zhao et al., 2011; Li et al., 2014). Stomatal closing assays were performed immediately after the opening assays, in which leaves were transferred to closing buffer containing 10 mM MES at pH 6.15 with or without the following treatments, as indicated: 20 μM ABA and 10 μM oryzalin (Kang et al., 2002; Jiang et al., 2014). NIS-Elements AR version 3.2 software was used for stomatal aperture measurements.

### Confocal Microscopy and Quantification of Microtubule (MT) Variations

Confocal microscopy was performed using a Nikon Eclipse C90i system. Images were taken at room temperature with a Plan Fluor 60× oil objective (numerical aperture of 1.4, excitation wavelength 488 nm and an emission wavelength of 516 nm). Z-stacks of 3–20 slices were collected of the cortical layer of guard cells (ensuring exclusion of any nuclear signal) and used for subsequent quantification. Any guard cell displaying only puncta and thus having a MT filament number of 0 was excluded from all quantitative analysis, unless otherwise noted. The number of filaments was determined by creating maximal intensity projections in ImageJ, drawing a line through the middle of each guard cell, generating a line profile, and then counting the number of discernable peaks (Eisinger W. R. et al., 2012; Li et al., 2014).

Occupancy was quantified using ImageJ, as previously described (Higaki et al., 2010; Akita et al., 2018). Briefly, occupancy was determined as follows: guard cells were rotated such that every image analyzed was facing the same orientation, all guard cells were individually isolated, a threshold was set in order to define MT filaments, and the ImageJ macro plug-in for density was applied to obtain pixel number. Occupancy was then defined as this pixel number divided by the area of the image.

The mean angular difference was quantified using Image J, as previously described (Yoneda et al., 2007; Higaki et al., 2010; Akita et al., 2015; Higaki, 2017) and was calculated as outlined in Supplementary Figure 1.^1^ First, a maximum intensity projection was created from the acquired z-stacks, and then the maximum intensity projection of the stoma was separated into individual guard cells. Using the freehand selection tool, the guard cell was outlined to create an ROI, which was subsequently added to the ROI manager. Prior to measuring the cell medial axis (angle), measurements were adjusted by selecting the “Fit Ellipse” option under “Set Measurements.” The cell medial axis was calculated in the ROI manager for each guard cell. The LPX Filter2d plugin, using the filter “lineFilters” and the linemode “lineExtract,” was used to skeletonize each guard cell. The default settings for “lineExtract” were used (giwslter = 5, mdnmsLen = 15, shaveLen = 5, delLen = 5, preGauss = -1). To remove background outside of the skeletonized guard cell, the color picker tool was selected and set to black. The image was then inverted and the area outside of the ROI was filled in. The average theta for each guard cell was calculated using the LPX Filter2d plugin, using the filter “lineFilters” and the linemode “lineFeature.” The mean angular difference was calculated by subtracting the angle of the cell medial axis over the horizontal (close to 90°) from the average theta and taking the absolute value.

### Statistics

**FIGURE 1:**
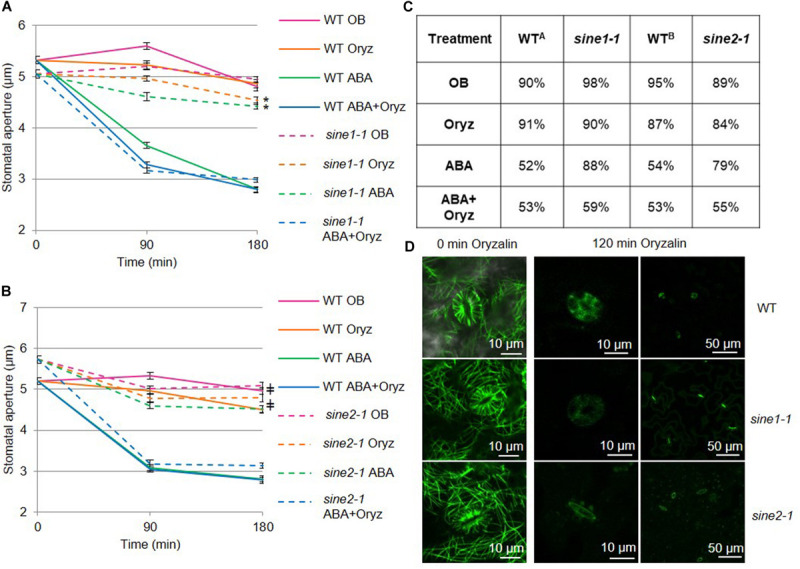
Disrupting microtubule organization in *sine1-1* and *sine2-1* mutant lines alters ABA-induced stomatal closure. Stomatal closure was monitored over a 3 h incubation time in the presence and absence of ABA and microtubule (MT) disrupting drugs. Buffers with and without 20 μM ABA and 10 μM of the MT depolymerizing drug oryzalin (oryz); **(A)** WT and *sine1-1*; **(B)** WT and *sine2-1*. **(C)** Stomatal aperture values converted to percentages. Starting apertures are set to 100% (fully open) and compared to apertures at the end of each assay, with smaller percentages meaning increased closure. WT^A^ indicates values for WT in Figure 1A. WT^B^ indicates values for WT in Figure 1B. **(D)** WT, *sine1-1*, and *sine2-1* expressing the fluorescently tagged MT marker GFP-MAP4 were treated with oryzalin under the same conditions. The lower magnification images on the right show stomatal pore autofluorescence. Symbols denote statistical significance as determined by Student’s *t*-test, with *P* < 0.001. *sine1-1 oryz vs. *sine1-1* ABA+Oryz; ^‡^*sine1-1* ABA vs. *sine2-1* ABA+Oryz. All data are mean values ± SE from three independent experiments with *N* ≥150 stomata.

The number of guard cells analyzed for each line, in all figures, is ≥38, except for Figure 1, which has ≥150 stomata. Error bars represent the standard deviation of means. Asterisks or symbols denote statistical significance after Student’s *t*-test as indicated.

## Results

### Functional Interaction Between *sine* Mutants and the Microtubule (MT) Cytoskeleton

Cortical MTs undergo a distinct reorganization during stomatal opening and closing, with depolymerization or stabilization of cortical MTs shown to influence stomatal dynamics (Fukuda et al., 1998; Yu et al., 2001; Lahav et al., 2004; Eisinger W. et al., 2012; Eisinger W. R. et al., 2012; Jiang et al., 2014; Khanna et al., 2014). Here, oryzalin, which causes MT depolymerization by binding to tubulin dimers and preventing MT assembly (Hugdahl and Morejohn, 1993), was used to test if the previously characterized *sine1-1* and *sine2-1* mutants functionally interact with MT depolymerization during ABA-induced stomatal closure.

Opening assays were performed prior to closing to ensure maximal opening of all stomata and the epidermal peels from rosette leaves were used for imaging, as described previously (Biel et al., 2020). Opening buffer (OB, see Materials and Methods) was used as a control throughout the entire assay and resulted in minimal stomatal closure in WT, *sine1-1*, and *sine2-1* plants (Figures 1A,B). Exogenous application of 20 μM ABA induced closure in WT but not in *sine1-1* and *sine2-1*, as previously reported (Figure 1; Biel et al., 2020). In WT, addition of 10 μM oryzalin was neither able to induce stomatal closure nor, when added with 20 μM ABA, did it enhance ABA-induced stomatal closure (Figures 1A,B). Oryzalin did not induce stomatal closure in *sine1-1* and *sine2-1*, however, closure comparable to WT was seen in the mutants in the presence of oryzalin and ABA.

In order to account for different starting apertures for each genetic background, percent stomatal closure after 180 min of ABA exposure was also calculated (Figure 1C). In WT exposed to ABA and oryzalin, aperture width was reduced to 53% compared to 91% with oryzalin alone (WT^*A*^), with similar values seen for *sine1-1* and *sine2-1*. WT^*A*^ closure was similar between the control OB treatment and oryzalin, 90 vs. 91%, respectively, with similar comparisons seen for *sine* mutants. As a control for the oryzalin treatment, oryzalin was used under the same conditions on WT and *sine1-1* expressing the fluorescently tagged MT marker GFP-MAP4 (Figure 1D). At 0 min, before addition of oryzalin, cortical MT filaments in guard cells and adjacent pavement cells were seen. After 120 min of oryzalin exposure, no MT filaments were visible in WT, *sine1-1*, and *sine2-1* (Figure 1D). In this context, filaments were defined as any visual filament that is GFP-labeled and no distinction was made between individual filaments or bundles.

Data from Figures 1A,B were collected independently. To directly compare the effects of oryzalin and ABA on *sine1-1* and *sine2-1*, a subset of data was also obtained concurrently for the two mutants (Supplementary Figure 2). Addition of ABA and oryzalin induce closure in *sine1-1* and *sine2-1* mutants to similar degrees and both *sine* mutants have similar lack of closure to oryzalin alone. Together, these data suggest that depolymerizing MTs can overcome the defect in ABA-induced stomatal closure caused by lack of SINE1 or SINE2.

### Guard Cell MT Organization, as Visualized by GFP-MAP4, Is Altered in *sine1-1* and *sine2-1*

**FIGURE 2:**
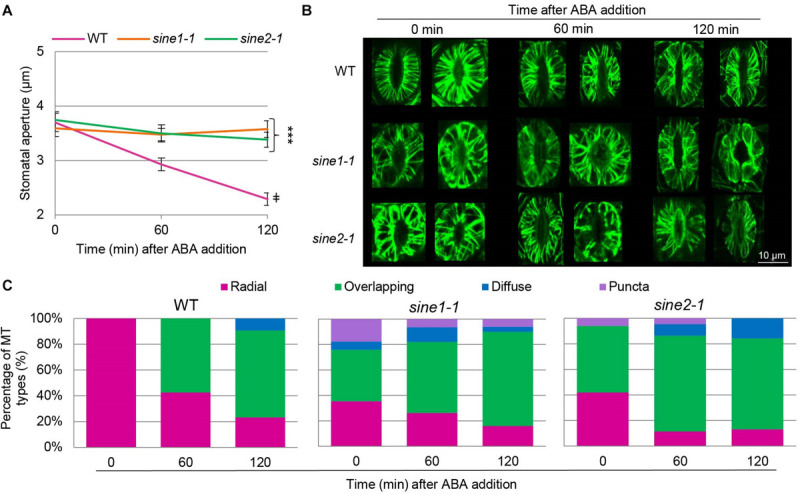
MT organization is altered in *sine* mutants. ABA-induced stomatal closing assays were used here as described in section “Materials and Methods.” WT, *sine1-1*, and *sine2-1* crossed with GFP-MAP4 were used. **(A)** Stomatal apertures for all three lines were monitored over the 3 h assay as a control for stomatal dynamics in GFP-MAP4 transgenic lines. Symbols denote statistical significance as determined by Student’s *t*-test, with ***P < 0.0005 for *sine1-1* and *sine2-1* vs. WT at 120 min and ^‡^*P* < 0.0005 for WT 0 min vs. WT 120 min. All data are mean values ± SE from three independent experiments, with N ≥ 26 stomata. **(B)** Representative images of MT organization at 0, 60, and 120 min after addition of ABA. Images shown are maximal intensity projections of the guard cell cortex. **(C)** MT filament patterns were grouped into several categories for WT, *sine1-1*, and *sine2-1*.

To visualize the cortical MT cytoskeleton, GFP-MAP4 was crossed with *sine1-1* and *sine2-1* (see section “Materials and Methods”) and F2 lines homozygous for *sine1-1* or *sine2-1* and homozygous or heterozygous for GFP-MAP4 were used for confocal imaging. The cortical layer of guard cells of rosette leaves from 3 to 4 weeks old long-day plants was imaged. Figure 2A shows that GFP-MAP4-expressing WT undergoes ABA-induced stomatal closure, while *sine1-1* and *sine2-1* do not, indicating that the marker did not affect stomatal dynamics under these conditions. WT, *sine1-1*, and *sine2-1* GFP-MAP4 transgenic plants were subjected to ABA and MTs were imaged at 0, 60, and 120 min after ABA addition. Representative images of maximal intensity projections of the guard cell cortex are shown in Figure 2B. In WT guard cells at 0 min, MTs appear uniformly and radially stacked with little to no crisscrossed or overlapping filaments. After 60 min of ABA exposure, MTs became dispersed and overlapping MT filaments became apparent. At 120 min, this dispersion and overlapping of MT filaments was even more prevalent. Conversely, MT filaments of *sine1-1* and of *sine2-1* showed differential MT organization with exposure to ABA (Figure 2). At 0 min, before addition of ABA, *sine1-1* guard cells showed larger gaps between filaments, more longitudinal filaments, overlapping filaments, and truncated filaments (that did not reach from dorsal to ventral wall; e.g., Figure 2B, middle row). After 60 min of ABA exposure, much of the same patterning was observed with an increase in the number of overlapping filaments. By 120 min ABA exposure, most MT filaments were in an intermediary state, displaying both overlapping and longitudinal filaments. Less frequently observed *sine1-1* patterns included: WT-like MT organization (Supplementary Figure 3A), intermediary MTs (Supplementary Figure 3B), occasional puncta in pavement cells (Supplementary Figure 3C), puncta in guard cells (Supplementary Figures 3D,E), and diffuse states (Supplementary Figure 3F). Lastly, *sine2-1* MTs at 0 min appeared to be thicker, displayed irregular gaps, and were overlapping (Figure 2B bottom row; Supplementary Figure 3G), which continued to increase similarly to WT and *sine1-1* with increased exposure to ABA (Figure 2B, bottom row). Less frequently observed *sine2-1* patterns included puncta patterning in pavement cells (Supplementary Figure 3H) and guard cells that were completely void of GFP signal (Supplementary Figures 3I–L).

The filament patterns were grouped into several categories, as adapted and expanded from Eisinger W. R. et al. (2012): radial, defined as organized and closely arrayed MT filaments traversing from the ventral to the dorsal guard cell wall; overlapping, defined as the presence of any irregular MT filament pattern, such as large gaps between filaments, crisscrossed filaments, and longitudinal or truncated filaments; diffuse, defined as consisting mostly of uniform fluorescence with few to no distinct filaments; and puncta, defined as distinct speckles. Images were visually scored and sorted into these four groups (Figure 2C). In WT, all guard cells had 100% radial MT organization (Figure 2C, Left panel). At 60 min, WT MT filaments were predominately overlapping (58%) while the remaining were still radial (42%). At 120 min, the distribution was 23% radial, 67% overlapping, and 10% diffuse. In contrast, already at 0 min, *sine1-1* had 35% radial, 41% overlapping, 6% diffuse filaments, and 18% puncta. This shifted at 60 min to 26% radial, 56% overlapping, 12% diffuse, and 6% puncta. By 120 min ABA exposure, most MT filaments were in an intermediary state, displaying overlapping and longitudinal filaments and quantified as: 16% radial, 74% overlapping, 4% diffuse, and 6% puncta. For *sine2-1*, patterns at 0 min were 42% radial, 52% overlapping, 0% diffuse, and 6% puncta; at 60 min, 11% radial, 75% overlapping, 9% diffuse, and 5% puncta; and at 120 min, 13% radial, 71% overlapping, 16% diffuse, and 0% puncta.

Overall, WT guard cells began in an organized state and, upon ABA perception, MT filaments were reorganized into an intermediary state largely lacking radial filaments. Conversely, *sine* mutants underwent less MT reorganization with exposure to ABA, with MT filaments already in an intermediary state when fully open.

### Quantitative Analysis of ABA-Induced Guard Cell MT Reorganization and the Effects of *sine* Mutants

**FIGURE 3:**
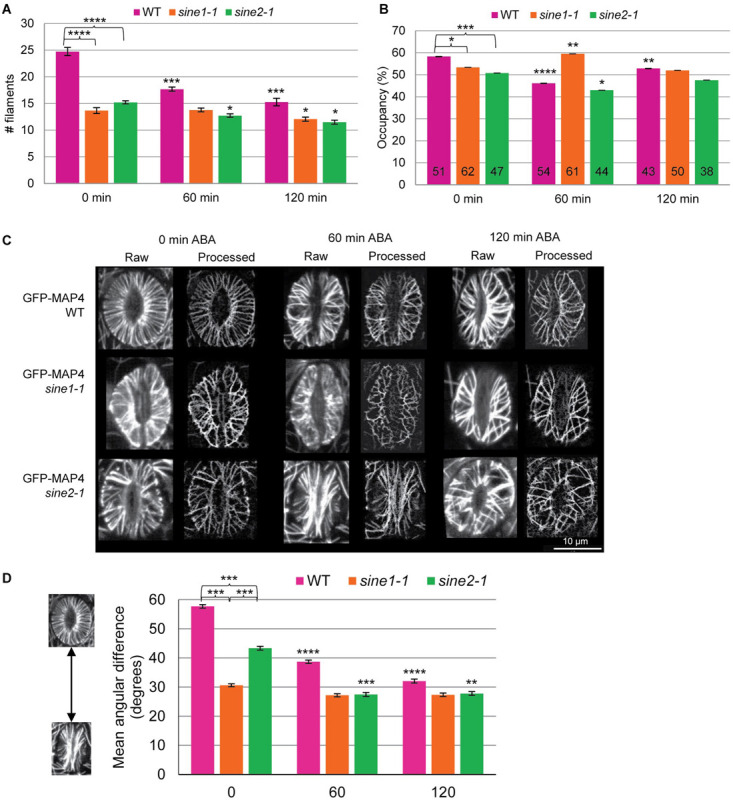
MT reorganization during ABA-induced stomatal closure is impaired in *sine* mutants. Quantitative analysis of MT patterns described in Figure 2. Three methods were employed for this analysis. **(A)** MT filament numbers in WT, *sine1-1*, and *sine2-1* guard cells. **(B)** Occupancy (density) of MT filaments in WT, *sine1-1*, and *sine2-1*. **(C)** Representative images demonstrating the skeletonization process. “Raw” designates original images collected through confocal microscopy, while “Processed” images are the skeletonized image generated in ImageJ. **(D)** The mean angular difference of MT filaments. All data are mean values ± SE from three independent experiments. Symbols at 0 min timepoints denote statistical significance as determined by Student’s *t*-test: *P < 0.05; ***P < 0.0005. Symbols at 60 and 120 min timepoints are compared to their 0 min timepoint counterparts with statistical significance as determined by Student’s paired *t*-test: *P < 0.05; **P < 0.005; ***P < 0.0005; ****P < 0.00005.

To quantify the differences in MT organization during ABA-induced stomatal closure, we employed three parameters: MT filament number (Figure 3A), occupancy (Figure 3B), and the mean angular difference (Figures 3C,D). These parameters have been used previously to quantify cytoskeletal changes in guard cells, for both actin and MTs (Yu et al., 2001, 2020; Higaki et al., 2010; Eisinger W. et al., 2012; Eisinger W. R. et al., 2012). MT filament number is a means of determining bundling in guard cells by counting the number of visible MT structures in a single guard cell (Yu et al., 2001, 2020; Eisinger W. R. et al., 2012). A visible MT structure was defined as a MT or MT bundle that could not be further optically resolved into finer structures. Any guard cell displaying only puncta and thus having a MT filament number of 0 was excluded from all quantitative analysis in Figure 3. WT guard cells displayed significantly more MT filaments compared to *sine1-1* or *sine2-1* (*P* < 0.0005) throughout the course of the assay, with the most notable difference seen at 0 min (Figure 3A). MT filament number was similar between *sine1-1* and *sine2-1* (*P* > 0.05) for each time point. Furthermore, WT also displayed the highest statistically significant changes in MT filament number during ABA-induced stomatal closure, as seen when comparing values at 60 and 120 min to that of the starting values (*P*-values from Student’s *t*-test shown in Supplementary Table 1). Thus, WT MTs were most responsive to ABA while *sine1-1* and *sine2-1* had much less change in MT filament number with addition of ABA.

Occupancy (displayed in %) was used here to measure the density of MT filaments within guard cells. Higher occupancy represents more space being occupied by MTs (as measured by increased fluorescence signal within the area of the guard cell). WT guard cells had highest occupancy at 0 min (Figure 3B). Occupancy then decreased at 60 min after ABA exposure before rising back up to an intermediate level by 120 min. Loss of SINE1 reversed this pattern, with *sine1-1* displaying its highest occupancy at 60 min and lower occupancy at 0 and 120 min. Meanwhile, loss of SINE2 resulted in occupancy changes similar to that of WT but the overall occupancy value was lower at each time point. As noted with MT filament number, *sine* mutants displayed less overall change in occupancy after ABA exposure compared to WT (Figure 3B).

Lastly, mean angular difference was used to quantify MT orientation, in which the average angles of each MT filament relative to the stomata cell medial axis was measured and displayed in degrees (see section “Materials and Methods,” Supplementary Figure 1, and Higaki, 2017). Briefly, maximum intensity projections of individual guard cells were vertically arrayed, isolated, and the cell medial axis was defined. The mean angular difference was measured between microtubule pixel pairs and the nearest segments of the cell medial axis in the processed images (Higaki et al., 2010). An angle closer to 90° represents more transverse filaments, while an angle closer to 0° indicates more longitudinal filaments. Representative images are shown in Figure 3C for WT, *sine1-1*, and *sine2-1* for each time point during the ABA-induced stomatal closure assay and the quantification is shown in Figure 3D. Each time point displays a single stoma in its raw, unprocessed state and in the processed state. The processed image is a black and white skeletonized image that contains only the MT filaments and no background fluorescence. The processed images are then used to obtain the mean angular difference. For WT at 0 min, which displays all radial filaments (see Figure 2), a mean angular difference of 58° was seen (Figure 3D). This is similar to Akita et al. (2018), which showed that stomata with fully radial MT filaments had a maximum mean angular difference of 60°. Mean angular difference decreased with increased ABA exposure. Similar to MT filament number, both *sine1-1* and *sine2-1* have significantly decreased mean angular difference values at every time point. Loss of SINE1 appears to have abolished any changes in MT re-organization, which resulted in similar mean angular difference values throughout the assay. Loss of SINE2 resulted in decreased mean angular difference but less so than compared to WT. Once again, a trend of significantly decreased ability to undergo WT-like transitions regarding MT organization during ABA-induced stomatal closure was seen here (Figure 2B, comparing 60 and 120 min timepoints to their counterpart values at 0 min). Together these data show that the KASH proteins SINE1 and SINE2 are required for radially arrayed MT reorganization in open guard cells as well as mediating changes in MT reorganization during stomatal closure.

## Discussion

We have previously reported that SINE1 and SINE2 play a role in stomatal dynamics in response to light, dark, and ABA, but not the bacterial elicitor flg22 (Biel et al., 2020). This ABA-hyposensitivity was attributed to impairments in Ca^2+^ and F-actin regulation. In this study, we link the ABA-hyposensitivity phenotype of *sine1-1* and *sine2-1* mutants to impairments in MT reorganization. We use the MT marker GFP-MAP4 to show that both proteins are involved in maintaining proper MT organization in open guard cells and mediating MT changes during ABA-induced stomatal closure.

Oryzalin has been previously used to influence stomatal dynamics in guard cells but mixed results have been reported (Thion et al., 1998; Marcus et al., 2001; Yu et al., 2001, 2020; Eisinger W. et al., 2012; Jiang et al., 2014; Khanna et al., 2014). Khanna et al. (2014) showed that oryzalin alone induced stomatal closure in WT Arabidopsis plants, whereas no change in stomatal apertures was observed with oryzalin alone by Jiang et al. (2014). These discrepancies may be due to the difference in the methods used. Although both studies use GFP-TUA6 to monitor MT organization, Khanna et al. (2014) uses 4–6 weeks old mature leaves while Jiang et al. (2014) uses 7-day old cotyledons. Using GFP-MAP4 and 3–4 weeks old mature leaves, our results show that WT plants exposed to oryzalin alone, like WT plants in opening buffer, did not close, while ABA-induced stomatal closure proceeded as expected and the combination of oryzalin and ABA did not lead to additional effects (Figures 1A,B). Thus, under our methods, oryzalin alone does not induce stomatal closure. It would be interesting to compare the impact of leaf age and MT-marker type (i.e., directly or indirectly tagging MTs) on MT organization to better understand these different experimental outcomes.

Interestingly, the combination of oryzalin and ABA rescued the previously reported defect in ABA-induced stomatal closure in *sine1-1* and *sine2-1*. Thus, in the absence of ABA signaling, oryzalin appears to have little to no impact on stomatal closure for both WT and *sine* mutants while in the presence of ABA, MT depolymerization appears to bypass a step normally requiring SINE1 and SINE2. This co-dependence on ABA and oryzalin suggests a crucial role for SINE proteins in mediating MT organization to allow for productive ABA-signaling and for stomatal closure.

It is currently not fully resolved how MTs are involved in stomatal closure. Some studies show that radial MTs in open guard cells become almost completely diffuse upon closure (Jiang et al., 2014; Yu et al., 2020), while others note that MT filaments are significantly decreased but, although some diffuse signal is seen, are still present (Eisinger W. et al., 2012; Khanna et al., 2014). The unifying characteristic is that MTs begin in a radial array in fully open guard cells and undergo a rearrangement (whether to a more bundled MT state or a depolymerized state), which is associated with stomatal closure. Utilizing the MT marker line GFP-MAP4, we found that MTs decrease in filament/bundle number but are still present after ABA-induced stomatal closure with little diffuse signal observed (Figure 2). A prior study observed that overexpression of GFP-MAP4 results in increased MT stability and inhibits stomatal dynamics (Marcus et al., 2001). However, Eisinger W. et al. (2012) found that GFP-MAP4 lines underwent H_2_O_2_-induced stomatal closure in addition to exhibiting MT rearrangement as shown by decreased numbers of MT filaments when closed as compared to open guard cells. We also show here that the addition of the GFP-MAP4 marker does not inhibit ABA-induced stomatal closing (Figure 2A). Additionally, MT rearrangement is still occurring in WT as seen by the significant decrease in MT filament number (Figure 3A), the change in MT filaments types (Figure 2C), and the significant change in mean angular variance (Figure 3D and Supplementary Table 1). Thus, although some stabilization and inhibition of depolymerization cannot be excluded, it is not sufficient to alter stomatal dynamics or inhibit MT rearrangement in WT guard cells.

Although discrepancies are seen with the role of MTs in guard cell closure, their role in opening is less uncertain. Cortical MTs within open guard cells are radially arranged and believed to be critical for maintaining an open stomatal state (Marcus et al., 2001). However, significantly fewer fully open guard cells of *sine1-1* and *sine2-1* had radially arrayed MTs after 2 h of induced opening with OB containing exogenous K^+^ and Ca^2+^ (40 vs. 100%, *sine* mutants and WT, respectively) (Figure 2C). The reason why *sine1-1* and *sine2-1* do not form fully radial arrays after 2 h of light treatment is currently not known. We recently showed evidence for opening impairments in *sine* mutants, which are only overcome in the presence of K^+^ and Ca^2+^. This induced opening may be linked to the ability of *sine1-1* and *sine2-1* to open guard cells without an intact radial MT patterning. The details behind the role of SINE1 and SINE2 in stomatal opening still need to be addressed.

**FIGURE 4:**
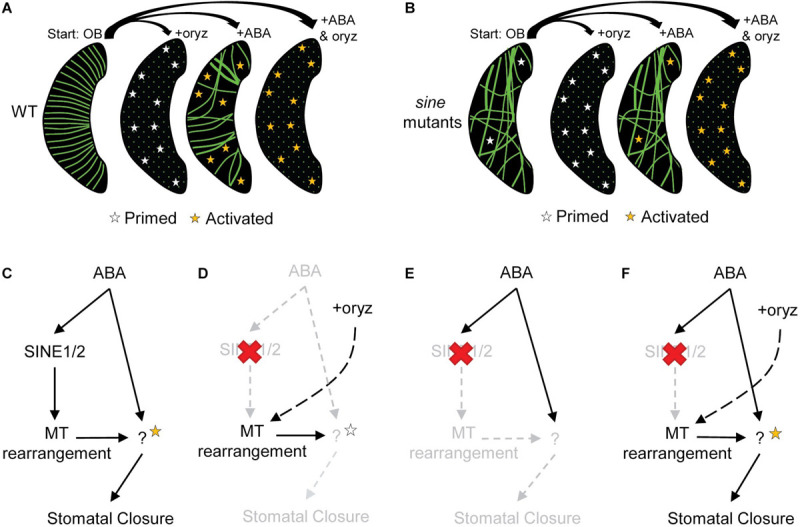
The role of MTs in ABA-induced stomatal closure. **(A,B)** Opening buffer (OB) represents fully opened guard cells before subsequent treatment with each of the following: oryzalin (oryz), ABA, and ABA+oryz. Guard cell depictions are illustrative outcomes for **(A)** WT and **(B)** *sine* mutants for each specified treatment group. Green lines represent MT filaments, and green dots representing diffuse MTs. **(A–F)** White stars represent primed ABA-specific downstream targets and orange stars represent activated ABA-specific downstream targets (see discussion). **(C–F)** ABA signaling model depicting the hypothetical role of SINE1 and SINE2 and stomatal closure outcomes under various treatments in **(C)** WT and **(D–F)** *sine1-1* and *sine2-1* mutants. **(C)** WT treated with ABA; **(D)** *sine* mutants with oryz treatment; **(E)** sine mutants with ABA treatment; **(F)** *sine* mutants with ABA+oryz cotreatment.

Based on the data presented here, we provide a working model for how SINE1 and SINE2, along with MTs, could be involved in ABA-induced stomatal closure (Figure 4). In this model, we suggest that MTs act to block unknown ABA-specific downstream targets (ABA targets). MT filaments would be precisely rearranged to relieve this inhibition and allow for ABA-induced stomatal closure to occur. This was shown for WT (Figures 4A,C), in which MTs began in a dense filamentous radial state and were arrayed in parallel. This parallel, dense array may increase MT occupancy within the guard cell, thus maximizing inhibitory MT interactions with ABA targets. After addition of ABA, MTs were reorganized in an intermediary state with fewer radial organized MTs and overall less MT filaments observed (likely by means of bundling). This would result in ABA targets to be released from their inhibitory state and be primed (white stars) for subsequent activation (orange stars) by ABA-signaling components. Thus, when MTs are depolymerized by oryzalin but ABA is not present, targets would be only primed, but not activated, and no stomatal closure would occur (Figures 4A,D).

With loss of SINE1 or SINE2 (Figures 4B,D–F), there was an overall decrease in MT filaments and there are fewer radially organized MTs. MTs are not undergoing reorganization and ABA targets would be inhibited (not primed for ABA activation), thus stomatal closure is blocked (Figures 4B,E). While oryzalin eliminates the presence of MT structures and allows for primed targets, there is no parallel ABA-signaling occurring to activate these targets and closure still does not occur (Figures 4B,D). Utilizing oryzalin to depolymerize MTs in *sine* mutants thus would bypass the MT reorganization defect, allowing for primed ABA targets that can be activated by ABA to induce stomatal closure (Figures 4B,F). Due to the unorganized MT array, it is possible that some ABA-specific downstream targets would not be inhibited by MTs (Figure 4B, white stars, OB) but the lack of coordinated MT rearrangement would result in sub-optimal levels of ABA-activated targets (Figure 4B, orange stars, ABA) that is not sufficient to induce closure.

One possible example for a MT-inhibited target could be a Ca^2+^-activated ion channel. ABA increases cytoplasmic Ca^2+^ levels (Munemasa et al., 2015) and several studies have correlated MT disorganization to impaired Ca^2+^ channel activity (Thion et al., 1996, 1998; Mazars et al., 1997; Khanna et al., 2014). Loss of SINE1 or SINE2 leads to changes in cytoplasmic calcium during ABA-induced stomatal closure (Biel et al., 2020). Neither SINE1 nor SINE2 colocalizes with MTs in guard cells and their role is therefore most likely indirect (Zhou et al., 2014). SINE1 and SINE2 mutants do, however, functionally interact with inhibitors of actin dynamics during stomatal closure (Biel et al., 2020). Actin-MT crosslinking factors are well-known, with perturbations in one cytoskeletal element influencing the organization of the other (He et al., 2020). Thus, the changes in MT organization observed here could be a result, perhaps in part, of impairments in actin reorganization (Biel et al., 2020). Alternatively, SINE1 and SINE2 might be involved in a currently unknown role of the guard cell nuclear envelope (NE) in MT organization. Plant MTOCs have been found to be associated with the NE in other cell types. Gamma-tubulin complex protein 3-interacting proteins (GIPs) are regulators of the recruitment of MT-nucleation complexes in Arabidopsis (Batzenschlager et al., 2015; Schmit et al., 2015; Batzenschlager et al., 2017). GIPs are found on both sides of the NE and thus can act as a MT organizing center at the NE. SINE proteins may interact with GIPs or other NE-associated proteins to regulate MT reorganization.

In summary, our observations show that the KASH proteins SINE1 and SINE2 are involved in the radial arrangement of MTs in open guard cells in addition to allowing MT rearrangement to occur. We propose that SINE1 and SINE2 act upstream of MT re-organization during ABA-induced stomatal closure, in parallel with ABA-downstream signaling, to coordinate stomatal closure. This study highlights the critical process of MT rearrangement to allow for ABA-induced stomatal closure and a role for KASH proteins in this process.

## Data Availability Statement

All datasets generated for this study are included in the article/Supplementary Material.

## Author Contributions

AB conceived project, designed experiments, performed experiments, analyzed and interpreted data, and wrote manuscript. MM analyzed and interpreted experiments and wrote manuscript. IM conceived project, supervised project, edited manuscript, and provided funding. All authors contributed to the article and approved the submitted version.

## Conflict of Interest

The authors declare that the research was conducted in the absence of any commercial or financial relationships that could be construed as a potential conflict of interest.

## Footnotes

1 https://lpixel.net/en/products/lpixel-imagej-plugins/

## Supplementary Material

Supplementary Figure 1 Description of Mean Angular Difference Calculation. **(A)** First, 0.2-μm-step serial optical sections of microtubules were obstained by confocal microscopy. Using the ImageJ plugin, a maximum intensity projection was created **(B)** and each guard cell was isolated **(C)**. Each guard cell was manually outlined to create an ROI **(D)**. The cell medial axis (angle) was calculated in the ROI manager for each guard cell **(E)**. The LPX Filter2d plugin (filter: lineFilters; linemode: lineExtract) was used to skeletonize each guard cell (F). The background outside the ROI was removed **(G)**. The LPX Filter2d plugin (filter: lineFilters; linemode: lineFeature) was used to calculate the average theta for each guard cell **(H)**. The mean angular difference was calculated by subtracting the guard cell angle **(E)** from the average theta **(H)** and taking the absolute value **(I)**.

Supplementary Figure 2 Oryzalin and ABA induced stomatal closure in *sine* mutants. Stomatal opening and closing assays were used here as described in methods. **(A)** Plants expressing GFP-MAP4 were evaluated for their ability to induce stomatal closure. Leaves were incubated in 20 μM ABA to induce closure; All data are mean values ± SE from three independent experiments. Symbols denote statistical significance as determined by Student’s *t*-test, with P < 0.001. ^∗^WT oryz vs. WT oryz+ABA; ^‡^*sine1-1* oryz vs. *sine1-1* oryz+ABA; ^¥^*sine2-1* oryz vs. *sine2-1* oryz+ABA. No statistical difference between WT oryz+ABA, *sine1-1* oryz+ABA, and *sine2-1* oryz+ABA at 180 min.

Supplementary Figure 3 Range of MT expression and patterning in *sine1-1* and *sine2-1.* MT patterns observed less frequently during ABA-induced stomatal closure in *sine1-1* **(A–F)** and *sine2-1* **(G–L)**. *sine1-1* guard cells exhibiting a WT-like MT organization **(A)** and an intermediate pattern **(B)**. Occasionally puncta were observed in *sine1-1* pavement cells **(C)** or in guard cells **(D,E)**. **(F)** *sine1-1* guard cells with diffuse MTs. **(G)** *sine2-1* guard cells with irregular gaps and overlapping MTs. **(H)** Puncta patterning in *sine2-1* pavement cells. **(I–L)** *sine2-1* guard cells where GFP-MAP4 appears “silenced” and lack a GFP signal.

Supplementary Table 1 Extended analysis of statistical significance for the data shown in Figure 2. Comparison is between time points for individual lines. Numbers shown are *P*-values from Student’s *t*-test with yellow boxes indicating highly significant differences (*P* < 0.005). **(A–C)** *t*-test values from data shown in Figure 2A (filament number) and Figure 2B (occupancy); **(A)** WT; **(B)** *sine1-1*; **(C)** *sine2-1*; **(D)** Mean angular difference for WT, *sine1-1*, and *sine2-1* from data shown in Figure 2D.
