# Supplementary material for: Arabidopsis KASH Proteins SINE1 and SINE2 Are Involved in Microtubule Reorganization During ABA-Induced Stomatal Closure
Source: Front Plant Sci. 2020 Nov 20;11:575573. doi: 10.3389/fpls.2020.575573 (PMC7722481; doi:10.3389/fpls.2020.575573)
Supplement: Supplementary Figure 1 — Description of Mean Angular Difference Calculation. (A) First, 0.2-μm-step serial optical sections of microtubules were obstained by confocal microscopy. Using the ImageJ plugin, a maximum intensity projection was created (B) and each guard cell was isolated (C). Each guard cell was manually outlined to create an ROI (D). The cell medial axis (angle) was calculated in the ROI manager for each guard cell (E). The LPX Filter2d plugin (filter: lineFilters; linemode: lineExtract) was used to skeletonize each guard cell (F). The background outside the ROI was removed (G). The LPX Filter2d plugin (filter: lineFilters; linemode: lineFeature) was used to calculate the average theta for each guard cell (H). The mean angular difference was calculated by subtracting the guard cell angle (E) from the average theta (H) and taking the absolute value (I). [file Data_Sheet_1.pdf]

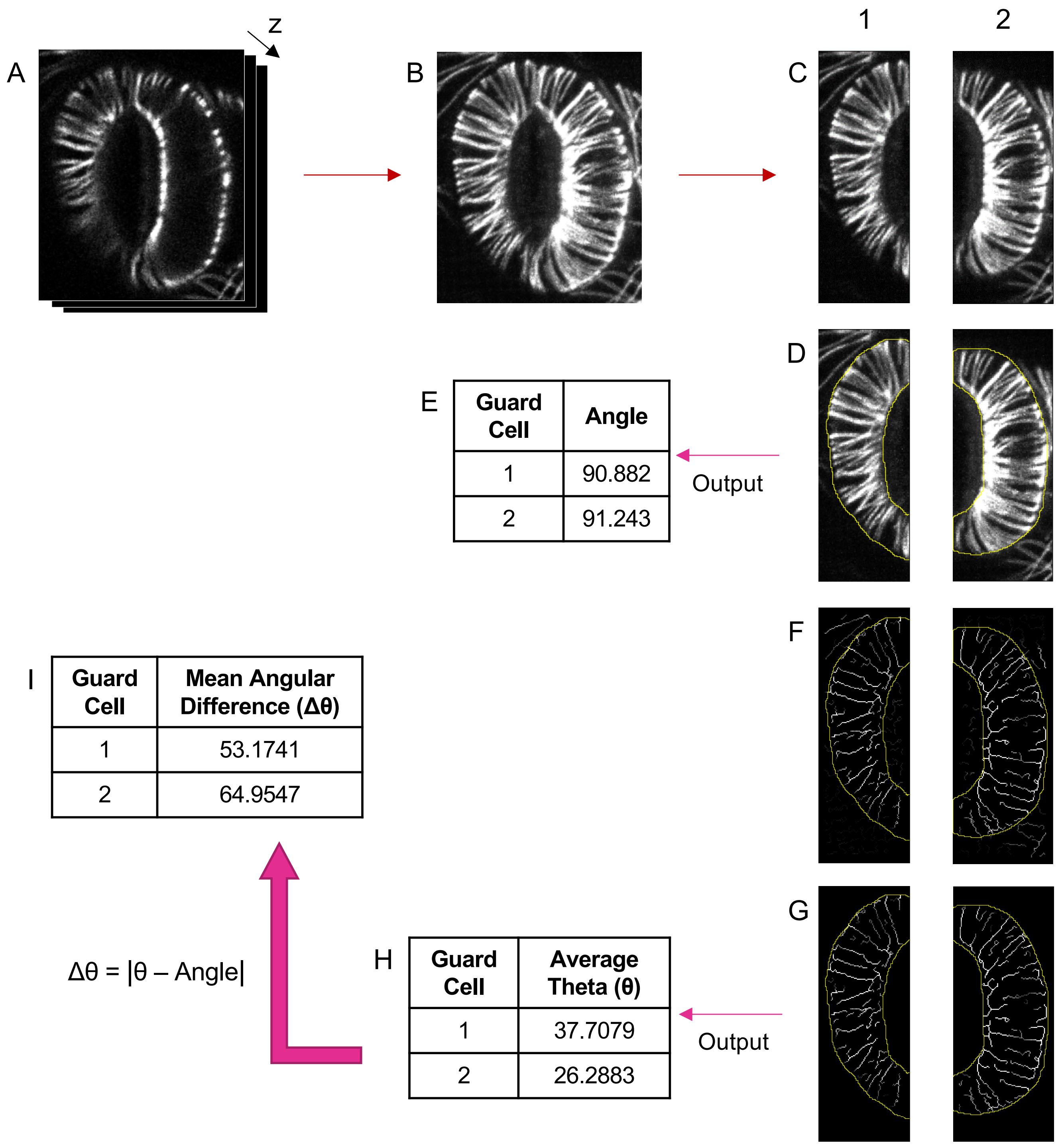

Guard Cell	Angle
1	90.882
2	91.243

Guard Cell	Average Theta (θ)
1	37.7079
2	26.2883

Guard Cell	Mean Angular Difference (Δθ)
1	53.1741
2	64.9547

Supplemental Figure 1: Description of Mean Angular Difference Calculation. (A) First, 0.2-μm-step serial optical sections of microtubules were obtained by confocal microscopy. Using the ImageJ plugin, a maximum intensity projection was created **(B)** and each guard cell was isolated **(C)**. Each guard cell was manually outlined to create an ROI **(D)**. The cell medial axis (angle) was calculated in the ROI manager for each guard cell **(E)**. The LPX Filter2d plugin (filter: lineFilters; linemode: lineExtract) was used to skeletonize each guard cell **(F)**. The background outside the ROI was removed **(G)**. The LPX Filter2d plugin (filter: lineFilters; linemode: lineFeature) was used to calculate the average theta for each guard cell **(H)**. The mean angular difference was calculated by subtracting the guard cell angle **(E)** from the average theta **(H)** and taking the absolute value **(I)**.

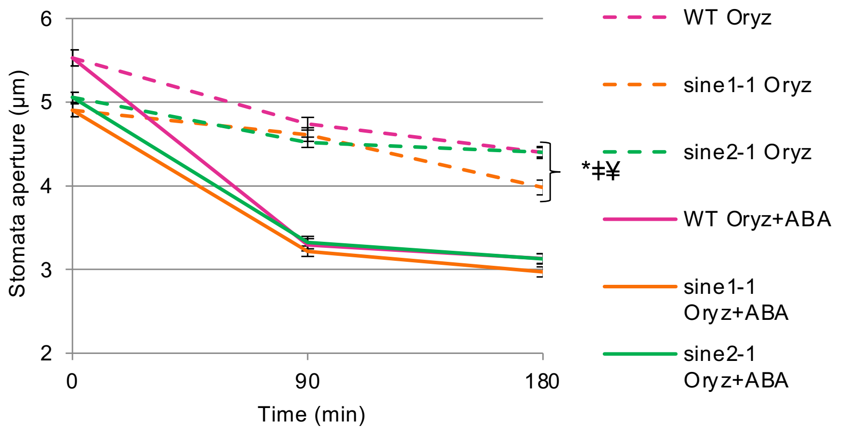

Supplemental Figure 2: Oryzalin and ABA induced stomatal closure. Stomatal opening and closing assays were used here as described in methods. **(A)** Plants expressing GFP-MAP4 were evaluated for their ability to induce stomatal closure. Leaves were incubated in 20 µM ABA to induce closure; All data are mean values ± SE from three independent experiments. Symbols denote statistical significance as determined by Student's t-test, with $P<0.001$. *: WT Oryz vs. WT Oryz+ABA; ‡: *sine1-1* oryz vs. *sine1-1* Oryz+ABA; ¥: *sine2-1* Oryz vs. *sine2-1* Oryz+ABA. No statistical difference between WT Oryz+ABA, *sine1-1* Oryz+ABA, and *sine2-1* Oryz+ABA at 180min.

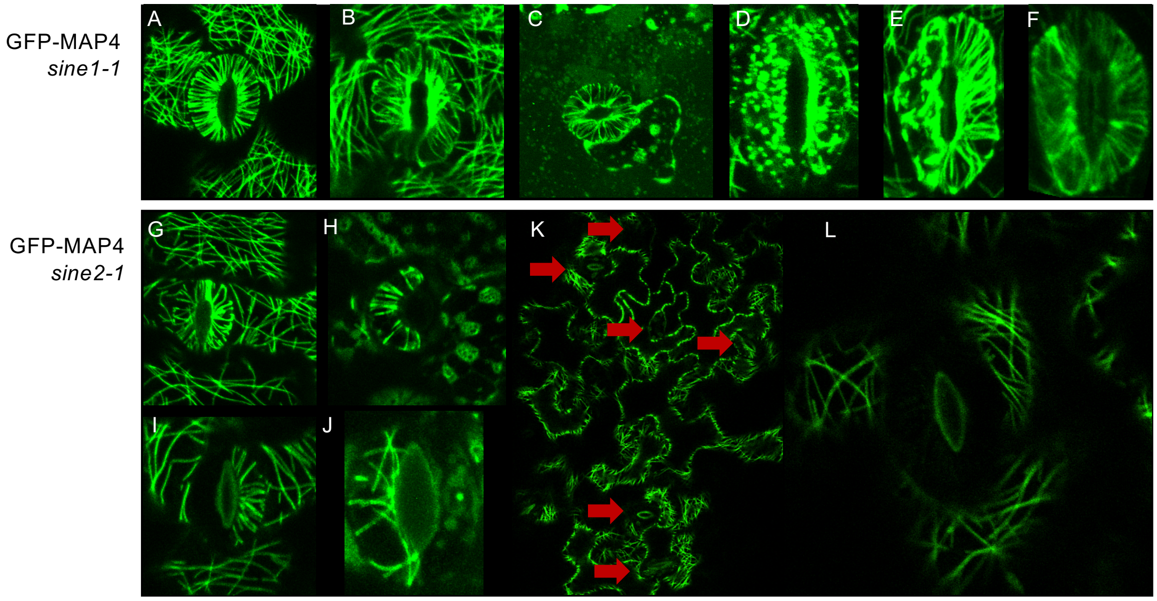

Supplemental Figure 3: Range of MT expression and patterning in *sine1-1* and *sine2-1.* MT patterns observed less frequently during ABA-induced stomatal closure in *sine1-1* **(A-F)** and *sine2-1* **(G-L)**. *sine1-1* GCs exhibiting a WT-like MT organization **(A)** and a disorganized pattern **(B)**. Occasionally puncta were observed in *sine1-1* pavement cells **(C)** or in GCs **(D,E)**. **(F)** *sine1-1* GCs with diffuse MTs. **(G)** *sine2-1* GCs with irregular gaps and overlapping MTs. **(H)** Puncta patterning in sine2-1 pavement cells. **(I-L)** *sine2-1* GCs where GFP-MAP4 appears “silenced” and lack a GFP signal.

Supplemental Table 1: Extended analysis of statistical significance for the data shown in Fig. 3. Comparison is between time points for individual lines. Numbers shown are P-values from Student's t-test with yellow boxes indicating highly significant differences (P<0.005). **(A-C)** t-test values from data shown in Fig. 3A (filament number) and 3B (occupancy); **(A)** WT; **(B)** *sine1-1*; **(C)** *sine2-1*; **(D)** Mean angular difference for WT, *sine1-1*, and *sine2-1* from data shown in Fig. 3D.

A

**WT**	**0 vs 60 min**	**0 vs 120 min**	**60 vs 120 min**
**Density**	9.3e-8	0.003	0.01
**# filaments**	0.0003	0.0008	0.1

B

sine1-1	**0 vs 60 min**	**0 vs 120 min**	**60 vs 120 min**
**Density**	0.006	0.5	0.0002
**# filaments**	0.3	0.01	0.2

C

sine2-1	**0 vs 60 min**	**0 vs 120 min**	**60 vs 120 min**
**Density**	0.02	0.4	0.1
**# filaments**	0.03	0.02	0.5

D

**MAD**	**0 vs 60 min**	**0 vs 120 min**	**60 vs 120 min**
**WT**	2.2e-7	1.7e-9	0.02
sine1-1	0.4	0.08	0.6
sine2-1	0.002	0.006	0.9
